# The Complex Interaction between Home Environment, Socioeconomic Status, Maternal IQ and Early Child Neurocognitive Development: A Multivariate Analysis of Data Collected in a Newborn Cohort Study

**DOI:** 10.1371/journal.pone.0127052

**Published:** 2015-05-21

**Authors:** Luca Ronfani, Liza Vecchi Brumatti, Marika Mariuz, Veronica Tognin, Maura Bin, Valentina Ferluga, Alessandra Knowles, Marcella Montico, Fabio Barbone

**Affiliations:** 1 Clinical Epidemiology and Public Health Research Unit, Institute for Maternal and Child Health—IRCCS “Burlo Garofolo,” Trieste, Italy; 2 Scientific Direction, Institute for Maternal and Child Health—IRCCS “Burlo Garofolo,” Trieste, Italy; 3 Department of Medical and Biological Sciences, University of Udine, Udine, Italy; 4 Department of Medical Sciences, University of Trieste, Trieste, Italy; 5 Institute of Hygiene and Clinical Epidemiology, University Hospital of Udine, Udine, Italy; Geisel School of Medicine at Dartmouth College, UNITED STATES

## Abstract

**Background:**

The relative role of socioeconomic status (SES), home environment and maternal intelligence, as factors affecting child cognitive development in early childhood is still unclear. The aim of this study is to analyze the association of SES, home environment and maternal IQ with child neurodevelopment at 18 months.

**Methods:**

The data were collected prospectively in the PHIME study, a newborn cohort study carried out in Italy between 2007 and 2010. Maternal nonverbal abilities (IQ) were evaluated using the Standard Progressive Matrices, a version of the Raven’s Progressive Matrices; a direct evaluation of the home environment was carried out with the AIRE instrument, designed using the HOME (Home Observation for Measurement of the Environment) model; the socioeconomic characteristics were evaluated using the SES index which takes into account parents occupation, type of employment, educational level, homeownership. The study outcome was child neurodevelopment evaluated at 18 months, with the Bayley Scales of Infant and Toddler Development Third Edition (BSID III). Linear regression analyses and mediation analyses were carried out to evaluate the association between the three exposures, and the scaled scores of the three main scales of BSID III (cognitive, language and motor scale), with adjustment for a wide range of potential explanatory variables.

**Results:**

Data from 502 mother-child pairs were analyzed. Mediation analysis showed a relationship between SES and maternal IQ, with a complete mediation effect of home environment in affecting cognitive and language domains. A direct significant effect of maternal IQ on the BSID III motor development scale and the mediation effect of home environment were found.

**Conclusions:**

Our results show that home environment was the variable with greater influence on neurodevelopment at 18 months. The observation of how parents and children interact in the home context is crucial to adequately evaluate early child development.

## Introduction

The development of the central nervous system is affected by the combination of genetic predispositions and social and physical environment. An adequate brain development depends on both prenatal and early postnatal experiences, and is the result of the continuous interaction between genetics and the context of life [1, 2].

In early childhood, one of the main factors affecting child cognitive development is the family environment, which includes both care and stimulation provided to children by the caregivers [3], and the socioeconomic status (SES) of the family [4]. During the first years of life, interaction with caregivers is crucial to ensure adequate psychosocial development of children and the absence of stimulation is associated with early social and cognitive disadvantage [5–7]. The parental socioeconomic status is a multidimensional construct based on several parameters, such as family income, material resources, education and occupation as well as related neighborhood and family characteristics [8]. The effects of SES on cognitive development of children are well known [9–11]. Children who grow up in families with lower SES are at increased risk of reduced psychological well-being and emotional and cognitive development. SES may affect neural development through a variety of different mediators, such as prenatal factors, parental care, cognitive stimulation, nutrition, stress, toxins and drugs exposure [10].

Maternal IQ is also associated with child neurocognitive development. Both a direct and indirect influence of maternal intelligence on child IQ has been suggested, the indirect influence being mediated by family income and home environment [12]. Other evidence suggests that the genetic effect on intelligence is similar in low- and high-SES families and that the greater variations in IQ in lower SES families are the result of children’s shared experiences [13].

A recent newborn cohort study showed the independent and positive influence of socioeconomic position, maternal IQ and home environment on child cognitive development [1] but, so far, only a limited number of studies has evaluated this complex relation providing an adequate and comprehensive adjustment for parental and environmental factors. In addition, more informative results could derive from the possibility of analyzing separately the different domains of child neurocognitive development (cognitive, linguistic and motor).

The aim of this study was to analyze the association of SES, home environment and maternal IQ (as proxy of heritability of intelligence) with child neurodevelopment, evaluated at 18 months with the Bayley Scales of Infant and Toddler Development Third Edition (BSID III) [14, 15]. For this purpose we used the data collected by a newborn cohort study (the PHIME project, NAC-II Cohort) [16] that allowed for adjustment for a wide range of potential explanatory variables.

## Materials and Methods

The PHIME project (Public health impact of long-term, low-level, mixed element exposure in susceptible population strata), NAC II cohort, had as its main objective, the evaluation of the association between low-level prenatal mercury (Hg) exposure through maternal fish consumption and child neurodevelopment in a coastal area of the Friuli Venezia Giulia region, in the north east of Italy. The study protocol and the results on the effects of prenatal Hg exposure have been published elsewhere [16, 17]. Briefly, the PHIME NAC II study was conducted at the Institute for Maternal and Child Health IRCCS Burlo Garofolo of Trieste (Italy), a third level hospital and one of the three pediatric research institutes recognized by the Italian Ministry of Health. The protocol of the PHIME study was approved on February 5^th^, 2007 by the Bioethics Committee of the Institute for Maternal and child Health IRCCS Burlo Garofolo. The enrolment of pregnant women started in April 2007. All Italian-speaking pregnant women ≥18 years of age living in the area of Trieste were contacted during pregnancy. A written informed consent was obtained. At the time of enrolment, on the occasion of ultrasound scans during pregnancy and at birth, biological samples (hair, blood, urine, and cord blood) were collected and two questionnaires containing information on food habits, socio demographic and health status were filled in by parents. The Raven’s Standard Progressive Matrices, a standardized intelligence test, were administrated to pregnant women (see below for details). Starting from 18 months after birth, a home visit was planned to evaluate the home environment using the AIRE instrument (see below); finally, in order to evaluate the child neurocognitive abilities at 18 months of age, the Bayley Scales of Infant and Toddler Development Third Edition (BSID III, explained in detail below) was administered during an *ad hoc* hospital visit.

For the purpose of the present evaluation the following data were used:

### Exposure variables

#### Measurement of maternal IQ

The Standard Progressive Matrices (SPM), a version of the Raven’s Progressive Matrices, is a test of nonverbal reasoning ability and general intelligence (IQ) [18]. Unlike other intelligence tests, SPM is independent from language, reading and writing skills. As a result of these characteristics, it minimizes cultural bias [19]. This standardized intelligence test evaluates the ability to think clearly and extract meaning out of events; it includes 60 items presented in 5 sets, with 12 items per set. The test consists of geometric analogy problems in which a series of geometric figures is presented with one entry missing, and the correct missing entry must be selected from a set of answer choices. Raw scores were converted to IQ scores which were used to measure performance compared with norms. The IQ score ranges between 62 and 128, with a mean of 100 (SD = 15) in the normative sample. The IQ index was then divided into quintiles in order to compare the lowest quintiles with the others. The test was administered by trained psychologists.

#### Measurement of home environment

The AIRE instrument, designed and validated by Capotorti [20], adapted from the HOME model (Home Observation for Measurement of the Environment) [21] to conform to Italian culture and social organization, was used. It includes an interview to the caregivers and an observation of parent-child interaction in the home context in order to evaluate four domains. A) communication and affective interaction between parents and child (i.e. mother spontaneously vocalizes to the child; the mother caresses or kisses the child); I) promotion of child autonomy (i.e. there are toys and activities for intellectual development, mother encourage the child to eat alone), R) respect for the child and implementation of rules (i.e. there is good compliance between parents about the home rules; caregivers do not shout at the child during the visit); E) emotional atmosphere (i.e. father provides some care-giving everyday; family visits or receives visits from relatives). The range of possible scores of the AIRE is 0–20 for each subscale. The AIRE visit lasted 30 to 40 minutes and was conducted by trained psychologists.

#### Measurement of socioeconomic characteristics

The SES index [22] was built taking into account the following parameters: parents’ occupation (1 = yes vs 0 = no), type of employment (1 = white collar vs 0 = blue collar following the Hollingshead classification), educational level (0 = up to middle school, 0.5 = high school, 1 = university degree), homeownership (1 = yes). On the basis of previous studies [22], these scores were summed (for type of employment and educational level the mean between parents was used) and the SES index obtained was then divided into quintiles in order to compare the lowest quintiles with the others.

### Measurement of children cognitive function (study outcome)

The Bayley Scales of Infant and Toddler Development (BSID III) [14, 15] was used. It is an individually administered scale that assesses the development of infants and toddlers between 1 and 42 months of age. The BSID III consists of 5 scales: 1) the cognitive scale (includes items on sensorimotor development, exploration and manipulation, object relatedness, concept formation, memory, and other aspects of cognitive processing); 2) the language scale (assesses preverbal behaviors, vocabulary development, vocabulary related to morphological development, understanding of morphological markers, social referencing and verbal comprehension, preverbal communication, vocabulary development, morpho-syntactic development, and includes two subscales evaluating receptive and expressive language); 3) the motor scale (assesses fine motor skill, perceptual motor integration, motor planning, motor speed, the movement of the limbs and torso, static position and dynamic movement, and includes two subscales evaluating fine motor and gross motor); 4) the social emotional scale; 5) the adaptive behavior scale. The cognitive, language, and motor scales were administered by trained psychologists, while the social-emotional and adaptive behavior questionnaires were self-administered by the parent or primary caregiver. Raw scores were converted to scaled scores. For cognitive scale and for each subscale of language and motor development the normative data had a mean of 10 and a standard deviation of 3. These scores were used to determine the child's performance (following the scoring instructions, the total values for language and motor scales were obtained adding the relative subscales values).

For the purpose of this analysis we only used the cognitive, language, and motor scales administered at 18 months of age.

### Potential explanatory variables

The following variables were also taken into consideration as potential explanatory variables of BSID III results: child's sex, birth weight, gestational age at birth, age of the mother at delivery, BMI before pregnancy, maternal mercury exposure measured in hair and in venous sample collected during pregnancy, house surface, mother living with partner, other children living in the house, maternal smoke, alcohol intake in pregnancy, dental visits, dental works, exclusivity of breastfeeding at 4 months, as defined by the WHO recommendations [23], child’s daycare attendance at 18 months.

### Statistical analysis

Only children born at or after 37 weeks of gestation, who underwent BSID III testing at 18±2 months and the AIRE home visit, were included in the analysis.

Continuous data are expressed as mean and standard deviation, categorical data as absolute frequencies and percentages. The association of the three main exposures and the other potential explanatory variables with the scaled score of the three main scales of the BSID III (cognitive, language and motor scale) were analyzed with multivariable linear regressions using a stepwise approach. A model was built for each of the BSID III scores. The three main variables were forced into the models, while the potential explanatory variables were retained if the p-value was less than 0.1. Data are presented as adjusted Beta regression coefficients and relative 95% Confidence Interval (95% CI). To avoid loss of statistical power in multivariate analyses, an imputation was carried out for the age of the mother (missing in 12 cases), assuming that missing occurred completely at random. No imputation was done for other variables, since the missing data were very limited (<0.01%).

#### Mediation and moderation analysis

Regression-based path analysis was used to estimate direct and indirect effects (path coefficients) of the three exposures under study (maternal IQ, SES and AIRE) on the main scales of BSID III (cognitive, language, motor). We decided to study the direct effect of the genetic proxy (maternal IQ) on child neurodevelopment, and to evaluate the mediation/moderation role of environmental exposures (family SES and home environment). Single or multiple mediation/moderation models were developed. For the indirect effects, 5,000 bootstrap samples were used for bias-corrected bootstrap confidence intervals.

#### Power analysis

We calculated that the 502 subjects enrolled would be sufficient for the purpose of the study based on the following assumptions: a) the probability of 80% to detect a relationship between the independent and the dependent variables at a two-sided 0.05 significance level; b) a mean value of BSID III cognitive scaled score (dependent variable) of 11; c) a standard deviation of BSID III scaled cognitive score of 1.6; d) a standard deviation of SES (independent variable) of 1; a true change in the BSID III scaled cognitive score of 0.2 unit per 1 unit change in SES.

Statistical analyses were performed using Stata software 11.2. The significance level was set to p<0.05 (two-tailed).

## Results

One thousand five hundred ninety five women were contacted during pregnancy; three hundred ninety-one resulted not eligible (due, for example, to language barriers or insufficient hair length for sampling). Of the remaining 1204 women, 900 (75%) were enrolled and 304 refused to participate. Seven hundred sixty-seven of the enrolled women (85%) remained in the study at delivery and 632 children underwent BSID III testing at 18 months. For 502 children it was also possible to carry out the home visit (55.8% of the initial cohort). More details on motivations that lead women to withdraw from the study are available elsewhere [24]. The socio-demographic characteristics of the final sample are presented in Table 1. The enrolled population consists mainly of Italian women with a mean age of 33.8 years (SD 4.4), with a relatively high education (University degree = 37.2%) and mostly employed (84.8%).

**Table 1:** Socio-demographic characteristics of 502 Italian children and their mothers.

Maternal age at delivery, years, mean (SD)		33.8 (4.3)
Maternal BMI before pregnancy, kg/m^2^, mean (SD)		22.7 (3.7)
Maternal country of birth, n (%) (n = 498)	Italy	463 (93.0)
	Other	35 (7.0)
Maternal marital status at delivery, n (%) (n = 496)	Married/living with partner	453 (91.3)
	Separated/divorced	13 (2.6)
	Single	30 (6.1)
Maternal educational level, n (%) (n = 500)	Elementary school	4 (0.8)
	Middle school	80 (16.0)
	High school	230 (46.0)
	University degree	186 (37.2)
Maternal occupational status, n (%) (n = 494)	Employed	419 (84.8)
	Housewife	40 (8.1)
	In search of employment	21 (4.2)
	Other	14 (2.8)
Maternal smoking, n (%) (n = 494)	Yes	38 (7.7)
	No	286 (57.9)
	No, ex-smoker	170 (34.4)
House ownership by the family, yes, n (%) (n = 495)		397 (80.2)
House surface, n (%) (n = 496)	<50 m^2^	35 (7.1)
	50–100 m^2^	334 (67.3)
	>100 m^2^	127 (25.4)
Number of children living in the house (including the newborn), n (%) (n = 486)	1	275 (56.6)
	2	170 (35.0)
	>2	41 (8.4)
Sex of the child, male, n (%)		266 (53.0)
Birth weight of the child, grams, mean (SD)		3431.0 (452.1)
Gestational age, weeks, n (%)	37	28 (5.6)
	38	82 (16.6)
	39	132 (26.7)
	40	135 (27.3)
	41	93 (18.8)
	42	25 (5.0)

Concerning study exposures, the mean value for the standardized IQ score was 118.9 (SD 11.2), a relatively high value, and 2.8 (SD 0.9) for family SES. The mean value obtained for total AIRE was 17.7 (SD 1.3) and as follows for the subscales: 1) for "communication between children and parents" 18.4 (SD 1.7); 2) for "promotion of autonomy" 18.7 (SD 1.3); 3) for "respect for the child and implementation of rules" 16.5 (SD 2.2); 4) for "emotional atmosphere" 17.1 (SD 2.3).

Mean scaled scores for BSID III (study outcome) were: 11.3 (SD 1.6) for the Cognitive scale, 19.2 (SD 2.9) for the Language scale and 20.4 (SD 1.8) for the Motor scale.

The results of the multivariate analysis evaluating the association between exposures and scaled scores of the BSID III are shown in Table 2.

**Table 2:** Multivariate analysis results.

			adj Beta	95% CI	P-value
**Cognitive scaled score** *	AIRE promotion of autonomy subscale		0.16	0.05 to 0.27	0.004
	Maternal IQ quintiles	I	-		
		II	0.25	-0.22 to 0.73	0.290
		III	0.37	-0.07 to 0.81	0.102
		IV	0.28	-0.12 to 0.69	0.173
		V	0.40	-0.10 to 0.91	0.118
	SES quintiles	I	-		
		II	0.06	-0.40 to 0.51	0.801
		III	0.19	-0.26 to 0.65	0.397
		IV	0.09	-0.38 to 0.56	0.705
		V	0.45	-0.03 to 0.93	0.065
**Language scaled score** ^#^	AIRE promotion of autonomy subscale		0.25	0.07 to 0.44	0.008
	Maternal IQ quintiles	I	-		
		II	0.78	0.03 to 1.53	0.041
		III	0.76	0.02 to 1.49	0.045
		IV	0.55	-0.16 to 1.26	0.131
		V	1.14	0.30 to 1.99	0.008
	SES quintiles	I	-		
		II	-0.75	-1.47 to -0.04	0.038
		III	-0.06	-0.77to 0.66	0.870
		IV	0.14	-0.67 to 0.95	0.733
		V	0.31	-0.46 to 1.08	0.432
**Motor development scaled score** ^§^	AIRE promotion of autonomy subscale		0.20	0.07 to 0.33	0.002
	Maternal IQ quintiles	I	-		
		II	0.38	-0.19 to 0.95	0.187
		III	0.57	0.01 to 1.13	0.045
		IV	0.50	-0.01 to 1.0	0.053
		V	0.93	0.36 to 1.50	0.001
	SES quintiles	I	-		
		II	-0.22	-0.67 to 0.23	0.334
		III	-0.04	-0.58 to 0.49	0.873
		IV	-0.48	-1.00 to 0.04	0.071
		V	-0.31	-0.81 to 0.19	0.228

Beta coefficients adjusted for:

* gestational age and dental visits

^#^ sex, exclusive breastfeeding at 4 months, birthweight < 2500gr, other children living in the house, daycare attendance at 18 months

^§^ exclusive breastfeeding at 4 months and maternal mercury exposure (hair and blood).

### Cognitive development

Among the three exposure variables, only the AIRE subscale "promotion of autonomy" was independently associated to the BSID III cognitive score, with an increase in cognitive score of 0.16 (95% CI 0.05–0.27) for each point of the subscale (Table 2).

Mediation analysis showed a direct effect of SES on cognitive development and a mediation effect of the AIRE "promotion of autonomy" subscale on this relation (see Table A and Figure A in S1 File). The model including the three exposure variables confirmed the relationship between SES and IQ and the role of AIRE as the main factor affecting child cognitive development. The indirect effect of AIRE and SES accounts for 34% of the total effect (p<0.05).

### Language development

Multivariate analysis showed that two exposure variables were independently related to language development (BSID III language scaled score): maternal IQ, in particular the highest quintile increased the score by 1.14 compared to the first quintile (95% CI 0.30–1.99), and the AIRE subscale "promotion of autonomy" (adjusted Beta 0.25, 95% CI 0.07–0.44) (Table 2).

Mediation analysis showed a direct effect of IQ on language development and a mediation effect of SES on this relation (see Table A and Figure A in S2 File). A direct effect of SES on language development, mediated by the AIRE "promotion of autonomy" subscale, was also showed. The model including the three exposure variables confirmed a relationship between SES and IQ and the role of AIRE as the main factor affecting child cognitive development. The indirect effect of AIRE and SES accounts for 29% of the total effect (p<0.05).

### Motor development

The highest quintiles of maternal IQ vs the lowest and the AIRE "promotion of autonomy" subscale resulted independently associated with the BSID III motor development scale (adjusted beta 0.93, 95% CI 0.36–1.50 and 0.20, 95% CI 0.07–0.33, respectively) (Table 2).

Mediation analysis confirmed the direct significant effect of IQ on the BSID III motor development scale and showed the indirect effect of the AIRE "promotion of autonomy" subscale: AIRE acts as mediator on the direct effect of IQ on motor development and accounts for 9.0% of the total effect. No direct or indirect effect of SES was found (see Table A and Figure A in S3 File).

No moderation effect of the three main exposures on the study outcomes was found.

## Discussion

Our analysis, which was carried out on data collected prospectively in a newborn cohort, confirms the complex relation between home environment, socioeconomic status, maternal IQ and early child neurocognitive development. In fact, the study results show that the three factors interact with each other to affect the different areas of early child development. Our results show a mediation effect of home environment (AIRE "promotion of autonomy" subscale) and family SES on the direct relation between maternal IQ (a proxy of heritability of intelligence) and cognitive and language development: the direct effect of maternal IQ was lost after considering the other two exposures. Conversely, a direct and indirect effect of maternal IQ on motor development was found. Home environment was the variable with the greatest influence on child neurodevelopment.

### Cognitive development

Our analysis shows that the main factor affecting child cognitive development at 18 months of age is home environment (in particular the "promotion of autonomy" subscale). The association between home environment and child cognitive development has already been reported: high scores on the HOME scale (the model from which the AIRE scale was derived) were associated with more advanced early cognitive development in children [25–27]. Also, evidence has been presented suggesting a strong relationship between quality of family context and child’s cognitive development [28].

A number of studies have addressed the relation between family socioeconomic status and child cognitive development [8–10]. SES may affect neural development through a number of different environmental factors, such as prenatal factors, parental care, cognitive stimulation, nutrition, parental stress, toxins or drugs exposure [10]. Recently, using magnetic resonance imaging, researchers have revealed a direct effect of SES on children's brain structure. In particular, lower SES was associated with smaller volumes of grey matter in several brain areas. These results, obtained in healthy children from developed countries, suggest that the structure of the brain can be affected by unfavorable environmental conditions even when these don’t reach extreme deprivation and stress [29]. The mediation analysis in this study confirms a direct effect of SES on cognitive score but the effect of home environment is most relevant.

The role of maternal IQ on child cognitive development is controversial. Conflicting evidence have been provided on this issue [1, 12, 13, 25, 30, 31], suggesting both direct and indirect influence of maternal intelligence on child IQ [1, 12] but also that this relationship could be influenced by home environment and shared experiences [13, 31]. Our results support the latter hypothesis, suggesting a major role of environmental factors and in particular of the home environment.

### Language development

Our mediation analysis suggests a direct relation between maternal intelligence and child language development and a mediation effect of SES on this relation. In turn, the direct relation between SES and language development is mediated by AIRE. The final model indicates that the main factor affecting child language is family environment. Previous studies have shown that SES can affect child language development through family environment. Hoff found that high-SES children between 12 and 24 month of age grew more, in terms of size of their productive vocabularies, than the mid-SES children. Properties of maternal speech that varied based on SES, fully accounted for this difference [32]. Hart and Risley have shown that environmental factors are associated with the quality of the parents’ sentences and, consequently, with the number of words learned during early infancy. In particular, in families with higher SES, parents use encouraging sentences more often than they do prohibitive ones, with a supposed effect on child development [33].

### Motor development

Mediation analysis showed that both home environment and maternal IQ affect child motor development. Also according to previous studies, home environment and parent-child interaction may have a crucial role in the development of motor function [34, 35]. The stimulation to manipulate toys and other age-appropriate objects and the encouragement to freely explore the home environment (both of which dimensions are associated to the AIRE "promotion of autonomy" subscale) result in better motor development, particularly during the early stages of childhood [36].

Our study shows a direct influence of maternal IQ on motor development. Evidence confirming this direct relationship is scanty [37]. An indirect effect of maternal intelligence, through a more stimulating child environment, has been shown [38].

No direct or indirect effect of SES on motor development was found in our study. Evidence on the role of SES on motor development are scarce and conflicting, showing a possible effect of social disadvantage in some cases [39], and a major role of other factors (i.e., home environment, parental style, hereditary) in others [36, 40, 41].

The main strength of the study is the adjustment for a wide range of possible explanatory variables including newborn characteristics (sex, birth weight, gestational age at birth, breastfeeding), mother characteristics (age of the mother at delivery, BMI before the pregnancy, smoke, alcohol intake), family characteristics (mother living with partner, other children living in the house, house size, daycare attendance at 18 months), exposure to heavy metals (mercury in blood and hair, dental interventions). The observation at home of mother-child pair interaction using the AIRE tool is expensive and time consuming. However, our results show that such observation is of paramount importance when studying child development, since the home environment influences the relationship between maternal IQ, SES and child development. Another strength of the study is the direct evaluation of the children’s cognitive function using the BSID III, administered by two trained psychologists in the course of a follow up visit in hospital. In a previous paper we demonstrated the very high interrater reliability of all BSID III scales evaluated, thereby ensuring that comparable results are obtained by the psychologists administering the test [42]. The use of the latest version of the BSID allowed for separate evaluation of linguistic and cognitive skills thus providing more reliable and informative data on the different developmental areas.

The main study limitation is the lack of adjustment for some variables potentially associated with the study outcomes, i.e. the father's IQ. As a result of this, we were unable to fully assess the effects of the genetic component on the neurodevelopmental outcomes. The transferability of the results may be limited since this study was carried out in a single north eastern Italian city. Furthermore, the "Italian language" inclusion criteria and the loss to follow up of women with low educational level [17] oversampled Italian highly-educated women with high IQ. It must be emphasized, however, that the high IQ score could be explained by the Flynn effect, which refers to the observed rise in IQ scores over time resulting in norms becoming obsolete [43], as indeed is the case for the Italian norms of the Raven test. Finally, the literature suggests that developmental predictors may change over time: i.e., maternal education influences language development at the age of 6 month, motor development at 18 month and all developmental dimensions at 36 month [44]. This effect makes it difficult to draw comparisons between our data and those available in literature, often collected at different ages, using different evaluation tests (i.e., older version of the Bayley test) and without a comprehensive control of possible predictors (i.e., few studies controlled for home environment). In the PHIME cohort, the age of 18 months was set by protocol based on the need for early assessment of enrolled children, but several authors have suggested it may be too early to adequately evaluate the neurocognitive development of children [45–48]. However, the cohort follow up will continue until the children have reached the age of seven, with an intermediate evaluation at 40 months, allowing for the evaluation of how developmental predictors may have modified in the course of time.

## Conclusions

Our results show that home environment, socioeconomic status and maternal intelligence are connected with each other in affecting the different domains of early child development. In particular, promotion of autonomy within the home environment seems to play a crucial role, in this age group, in all observed domains (cognitive, language and motor). Therefore, the observation of how parents and children interact in the home context, despite being expensive and time consuming, is essential to adequately evaluate early child development [49]. This finding may be useful also to plan early interventions aimed at ensuring adequate psychosocial development in children.

## Supporting Information

S1 File Results of mediation analysis for child cognitive development. (DOC)

S2 File Results of mediation analysis for child language development. (DOC)

S3 File Results of mediation analysis for child motor development. (DOC)
