# Supplementary material for: The Complex Interaction between Home Environment, Socioeconomic Status, Maternal IQ and Early Child Neurocognitive Development: A Multivariate Analysis of Data Collected in a Newborn Cohort Study
Source: PLoS One. 2015 May 21;10(5):e0127052. doi: 10.1371/journal.pone.0127052 (PMC4440732; doi:10.1371/journal.pone.0127052)
Supplement: S1 File — (DOC) [file pone.0127052.s001.doc]

**S1 File. Results of mediation analysis for child cognitive development**

**Table A**

|  | **path coefficients (95% CI)**  **model 1** | **path coefficients (95% CI)**  **model 2** | **path coefficients (95% CI)**  **model 3** |
| --- | --- | --- | --- |
| SESq→ AIREp | NA | 0.100 (0.024 – 0.176)* | NA |
| SESq →BSID cognitive | 0.113 (0.006 - 0.219)* | 0.118 (0.017 – 0.219)* | 0.096 (-0.011 – 0.202) |
| AIREp → BSID cognitive | NA | 0.176 (0.061 – 0.291)* | 0.159 (0.049 – 0.270)* |
| SESq → AIREp → BSID cognitive | NA | 0.018 (0.003 - 0.042^a^)* | NA |
| IQq → BSID cognitive° | 0.093 (-0.018 - 0.204) | NA | 0.086 (-0.024 -0.196) |
| IQq → SESq | 0.339 (0.252 - 0.426)* | NA | 0.340 (0.253- 0.428)* |
| IQq → AIREp | NA | NA | 0.078 (-0.006 – 0.161) |
| IQq → SESq → BSID cognitive | 0.038 (0.004 - 0.079 ^a^)* | NA | 0.033 (-0.003 – 0.074 ^a^) |
| IQq → AIREp → BSID cognitive | NA | NA | 0.012 (0.0001 – 0.036 ^a^)* |
| IQq → SESq → AIREp → BSID cognitive | NA | NA | 0.045 (0.007 - 0.092 ^a^)* |

°unadjusted coefficient 0.115 (95%CI 0.009 - 0.220), p=0.033

model 1: SES (IV), AIRE promotion of autonomy subscale (AIREp) (M1), BSID scaled cognitive score.

model 2: SES (IV), AIRE promotion of autonomy subscale (AIREp) (M), BSID scaled cognitive score.

model 3: model 1 + AIRE promotion of autonomy subscale (AIREp) as mediator (M2). Proportion of total effect that is mediated 0.34, p<0.05.

*p<0.05

^a^ bootstrapped bias corrected confidence interval

Legend: AIREp: AIRE, promotion of autonomy subscale; BSDI cognitive: Bayley Scales of Infant and Toddler Development, scaled cognitive score; IQq: maternal IQ, quintiles; NA: not applicable; SESq: family socioeconomic status index, quintiles.

**Figure A**

**Model 1**

SESq

IQq

BSID cognitive

0.093

0.113*

0.339*

**Model 2**

AIRE p

SESq

BSID cognitive

0.118*

0.176*

0.100*

**Model 3**

0.086

0.340*

0.096

0.078

0.159*

SESq

IQq

AIRE p

BSID cognitive

Legend:

Numbers reported beside arrows represent adjusted coefficients; * p<0.05

AIRE p: AIRE, promotion of autonomy subscale; BSDI cognitive: Bayley Scales of Infant and Toddler Development, scaled cognitive score; IQq: maternal IQ, quintiles; SESq: family socioeconomic status index, quintiles.
