# Supplementary material for: The Complex Interaction between Home Environment, Socioeconomic Status, Maternal IQ and Early Child Neurocognitive Development: A Multivariate Analysis of Data Collected in a Newborn Cohort Study
Source: PLoS One. 2015 May 21;10(5):e0127052. doi: 10.1371/journal.pone.0127052 (PMC4440732; doi:10.1371/journal.pone.0127052)
Supplement: S2 File — (DOC) [file pone.0127052.s002.doc]

**S2 File. Results of mediation analysis for child language development**

**Table A**

|  | **path coefficients (95% CI)**  **model 1** | **path coefficients (95% CI)**  **model 2** | **path coefficients (95% CI)**  **model 3** |
| --- | --- | --- | --- |
| SESq→ AIREp | NA | 0.100 (0.024 – 0.176)* | NA |
| SESq →BSID language | 0.186 (0.011 - 0.361)* | 0.219 (0.053 – 0.386)* | 0.165 (-0.010 – 0.340) |
| AIREp → BSID language | NA | 0.254 (0.064 – 0.443)* | 0.248 (0.059 – 0.437)* |
| SESq → AIREp → BSID language | NA | 0.025 (0.003 – 0.066^a^) | NA |
| IQq → BSID language | 0.188 (0.006 - 0.370)* | NA | 0.177 (-0.004 – 0.357) |
| IQq → SESq | 0.336 (0.250 - 0.422)* | NA | 0.338 (0.251 – 0.424)* |
| IQq → AIREp | NA | NA | 0.060 (-0.019 – 0.140) |
| IQq → SESq → BSID language | 0.062 (0.003 - 0.131)* | NA | 0.056 (-0.004 – 0.122 ^a^) |
| IQq → AIREp → BSID language | NA | NA | 0.015 (-0.003 – 0.054 ^a^) |
| IQq → SESq → AIREp → BSID language | NA | NA | 0.071 (0.068 – 0.142 ^a^)* |

°unadjusted coefficient 0.252 (95%CI 0.071 - 0.434), p=0.006

model 1: SES (IV), AIRE promotion of autonomy subscale (AIREp) (M1), BSID scaled language score.

model 2: SES (IV), AIRE promotion of autonomy subscale (AIREp) (M), BSID scaled language score.

model 3: model 1 + AIRE promotion of autonomy subscale (AIREp) as mediator(M2). Proportion of total effect that is mediated 0.29, p<0.05.

*p<0.05

^a^ bootstrapped bias corrected confidence interval

Legend: AIREp: AIRE, promotion of autonomy subscale; BSDI language: Bayley Scales of Infant and Toddler Development, scaled language score; IQq: maternal IQ, quintiles; NA: not applicable; SESq: family socioeconomic status index, quintiles.

**Figure A**

**Model 1**

SESq

IQq

BSID language

0.188*

0.186*

0.336*

**Model 2**

AIRE p

SESq

BSID language

0.219*

0.254*

0.100*

**Model 3**

SESq

IQq

AIRE p

BSID language

0.338*

0.165

0.060

0.248*

0.177

Legend:

Numbers reported beside arrows represent adjusted coefficients; * p<0.05

AIRE p: AIRE, promotion of autonomy subscale; BSDI language: Bayley Scales of Infant and Toddler Development, scaled language score; IQq: maternal IQ, quintiles; SESq: family socioeconomic status index, quintiles.
