# Supplementary material for: The Complex Interaction between Home Environment, Socioeconomic Status, Maternal IQ and Early Child Neurocognitive Development: A Multivariate Analysis of Data Collected in a Newborn Cohort Study
Source: PLoS One. 2015 May 21;10(5):e0127052. doi: 10.1371/journal.pone.0127052 (PMC4440732; doi:10.1371/journal.pone.0127052)
Supplement: S3 File — (DOC) [file pone.0127052.s003.doc]

**S3 File. Results of mediation analysis for child motor development**

**Table A**

|  | **path coefficients (95% CI)**  **model 1** |
| --- | --- |
| IQq → BSID motor | 0.160 (0.045 - 0.275)* |
| IQq → AIREp | 0.080 (-0.003 - 0.164) |
| AIREp → BSID motor | 0.189 (0.068 - 0.307)* |
| IQq → AIREp → BSID motor | 0.015 (0.0002 - 0.043^a^)* |

Adjusted for age at AIRE testing, birth weight and exclusive breastfeeding at 4 months.

Proportion of total effect that is mediated 0.09

*p<0.05

^a^ bootstrapped bias corrected confidence interval

Legend: AIREp: AIRE, promotion of autonomy subscale; BSDI motor: Bayley Scales of Infant and Toddler Development, scaled motor score; IQq: maternal IQ, quintiles.

**Figure A**

AIRE p

IQq

BSID motor

0.160*

0.189*

0.080

Legend:

Numbers reported beside arrows represent adjusted coefficients; * p<0.05

AIRE p: AIRE, promotion of autonomy subscale; BSDI motor: Bayley Scales of Infant and Toddler Development, scaled motor score; IQq: maternal IQ, quintiles
